# Characterization and identification of sources of rust resistance in *Triticum militinae* derivatives

**DOI:** 10.1038/s41598-024-59902-x

**Published:** 2024-04-24

**Authors:** Saikat Chowdhury, Shreshtha Bansal, Shailendra K. Jha, M. S. Saharan, Niranjana M., Raghunandan K., Manish K. Choudhary, Priyanka Agarwal, Niharika Mallick, Vinod

**Affiliations:** 1 Division of Genetics, ICAR-Indian Agricultural Research Institute, New Delhi, 110012 India; 2 Division of Plant Pathology, ICAR-Indian Agricultural Research Institute, New Delhi, 110012 India

**Keywords:** *T. militinae*, Leaf rust, Stripe rust, Seedling resistance, Adult plant resistance, Genetics, Plant sciences

## Abstract

*Triticum militinae* (2n = 4X = 28, A^t^A^t^GG), belonging to the secondary gene pool of wheat, is known to carry resistance to many diseases. Though some disease resistance genes were reported from *T. timopheevii,* the closest wild relative of *T. militinae,* there are no reports from *T. militinae.* Twenty-one *T. militinae* Derivatives (TMD lines) developed at the Division of Genetics, IARI, New Delhi, were evaluated for leaf and stripe rusts at seedling and adult plant stages. Eight TMD lines (6–4, 6–5, 11–6, 12–4, 12–8, 12–12, 13–7 and 13–9) showed seedling resistance to both leaf and stripe rusts while six TMD lines (7–5, 7–6, 11–5, 13–1, 13–3 and 13–4) showed seedling resistance to leaf rust but adult plant resistance to stripe rust and three TMD lines (9–1, 9–2 and 15) showed seedling resistance to leaf rust but susceptibility to stripe rust. Three TMD lines (2–7, 2–8 and 6–1) with adult plant resistance to leaf and stripe rusts were found to carry the known gene *Lr34/Yr18*. Ten TMD lines (7–5, 7–6, 9–1, 9–2, 11–5, 11–6, 12–12, 12–4, 12–8, and 15) with seedling resistance to leaf rust, showing absence of known genes *Lr18* and *Lr50* with linked markers requires further confirmation by the test of allelism studies*.* As not a single stripe rust resistance gene has been reported from *T. militinae* or its close relative *T. timpopheevii*, all the 8 TMD lines (6–4, 6–5, 11–6,12–4, 12–8, 12–12, 13–7 and 13–9) identified of carrying seedling resistance to stripe rust and 3 TMD lines (13–1, 13–3 and 13–4) identified of carrying adult plant resistance to stripe rust are expected to carry unknown genes. Also, all the TMD lines were found to be cytologically stable and thus can be used in inheritance and mapping studies.

## Introduction

Wheat (*Triticum aestivum* L.) is one of the most important cereal crops in the world and India. It serves as a major source of protein to lower- and middle-income nations and stands second to rice in meeting the calorie requirements. India stands second in terms of production after China. India produced 110.55 million tonnes of wheat during 2022–23, higher than the previous year^1^. Though India is seeing a trend of increase in wheat production every year, it is still having effort meeting the demands of an ever-growing population. The production and productivity of wheat is affected by several biotic and abiotic factors. Among biotic factors, wheat rusts caused by three *Puccinia species* (leaf rust or brown rust caused by *Puccinia triticina, s*tem rust or black rust by *Puccinia graminis* f. sp. *tritici* and stripe rust or yellow rust by *Puccinia striiformis* Westend) are known to cause significant yield losses to wheat worldwide. The impact of rust on yield reduction in wheat ranges from 10% under moderate to 65% under intense epidemics^2^. However, 100% loss can occur due to stripe rust if it occurs on susceptible cultivars under favourable climatic conditions^3,4^. So, breeding for genetic resistance in wheat for these diseases is of utmost importance. Till now, a total of 83 leaf rust resistance (*Lr*) genes, 63 stem rust resistance (*Sr*) genes and 86 stripe rust resistance (*Yr*) genes have been documented^5–9^. Apart from these, several QTLs for leaf and stripe rust resistance have been identified and documented. However, many of these designated genes became ineffective over the years due to the evolution of new virulent pathotypes against them. Among the commonly used major leaf rust resistance genes like *Lr1*, *Lr3*, *Lr9*, *Lr10*, *Lr13, Lr14a, Lr17, Lr19, Lr23, Lr24*, *Lr26* and *Lr28*, which exhibited all stage resistance, only *Lr24* remained effective against prevalent pathotypes in India; however, races virulent on *Lr24* have been reported from other parts of the world^10^. In the case of stripe rust, many previously deployed stripe rust resistance genes, such as *Yr9* and *Yr27,* became ineffective due to the evolution of virulent pathotypes^11–13^.

Wild and related species of wheat carry enormous genetic variability that can be used in breeding programmes to broaden the genetic base of cultivated wheat. About half of the genes have been transferred to wheat through alien introgression^14–17^. Many wild species belonging to secondary and tertiary gene pools carry abundant genes resistant to biotic and abiotic stresses. However, they are still untapped because of the difficulty in making wide crosses and getting fertile F_1_ seeds^16,18^. One such wild species, *Triticum militinae*, belonging to the secondary gene pool of wheat, carries enormous genetic variability for disease resistance. *T. militinae* is considered a spontaneous mutant of *T. timopheevii*^19^. However, another view is that it originated from an introgressive hybridization between *T. timopheevii* and *T. cathlicum* Nevski (*T. persicum* Vav.)^20^. *T. timopheevii* has immunity to many diseases, such as leaf rust, stripe rust, stem rust, powdery mildew, loose smut, karnal bunt and dwarf smut^21^. However, till now, not a single disease resistance gene has been reported from *T. militinae*. As such, five-leaf rust resistance genes (*Lr18*^22^, *Lr50*^23^, *LrTt1*^24^, *LrTt2*^25^ and *LrSelG12*^26^)*,* four stem rust resistance genes (*Sr36*, *Sr40*, *SrTt3* and *Sr37*)^27^ and three powdery mildew resistance genes (*Pm6*^28^, Pm27^29^ and *Pm37*^30^) were reported to be transferred from *T. timopheevii* to bread wheat. Breeders have always been interested in exploring this untapped wild species to identify useful resistance genes. At the Division of Genetics, IARI, New Delhi, the wheat group has developed 44 introgression lines (ILs), named TMD lines (*T. militinae* Derivatives). They were developed by crossing bread wheat lines, Chinese Spring and NI5439 with *Triticum militinae* acc. No. 117001 and backcrossing the F_1_s to Chinese Spring for three generations and further selfing of BC_3_F_1_ for seven generations. Earlier, Natraj et al. 2017 characterized three leaf rust resistance TMD lines (TMD6-4, TMD7-5 and TMD11-5) using SSR markers to identify the *T. militinae* introgression points in these three derivatives. In the current study, we have used 21 TMD lines (based on stability in their performance over the years) for screening at seedling and adult plant stages for leaf and stripe rusts. It gave us information on whether a particular derivative carries seedling or adult plant resistance or both. Also, the markers of known genes were used to screen these 21 TMD lines and to obtain initial knowledge of lines with unidentified sources of rust resistance.

## Results

### Cytological analysis

**Figure 1:**
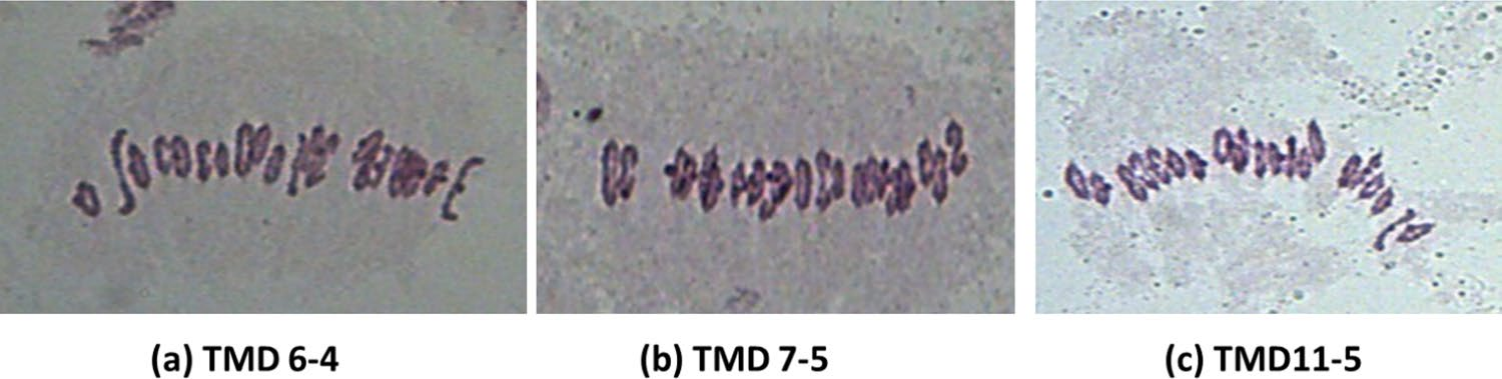
Meiotic analysis of some *T. militinae* derivatives showing 21 bivalents.

Cytological analysis of twenty-one TMD lines showed the presence of 42 chromosomes (21 bivalents) in all the derivatives during meiotic metaphase I. The presence of 21 bivalents indicates the cytological stability of these *T. militinae* derivatives and the absence of any numerical aberration in them. This study also indicates the suitability of these TMD lines for inheritance and mapping studies. Representative pictures showing 21 bivalents in some of the TMD lines are presented in Fig. 1.

### Identification of leaf rust-resistant *T. militinae* derivatives

**Figure 2:**
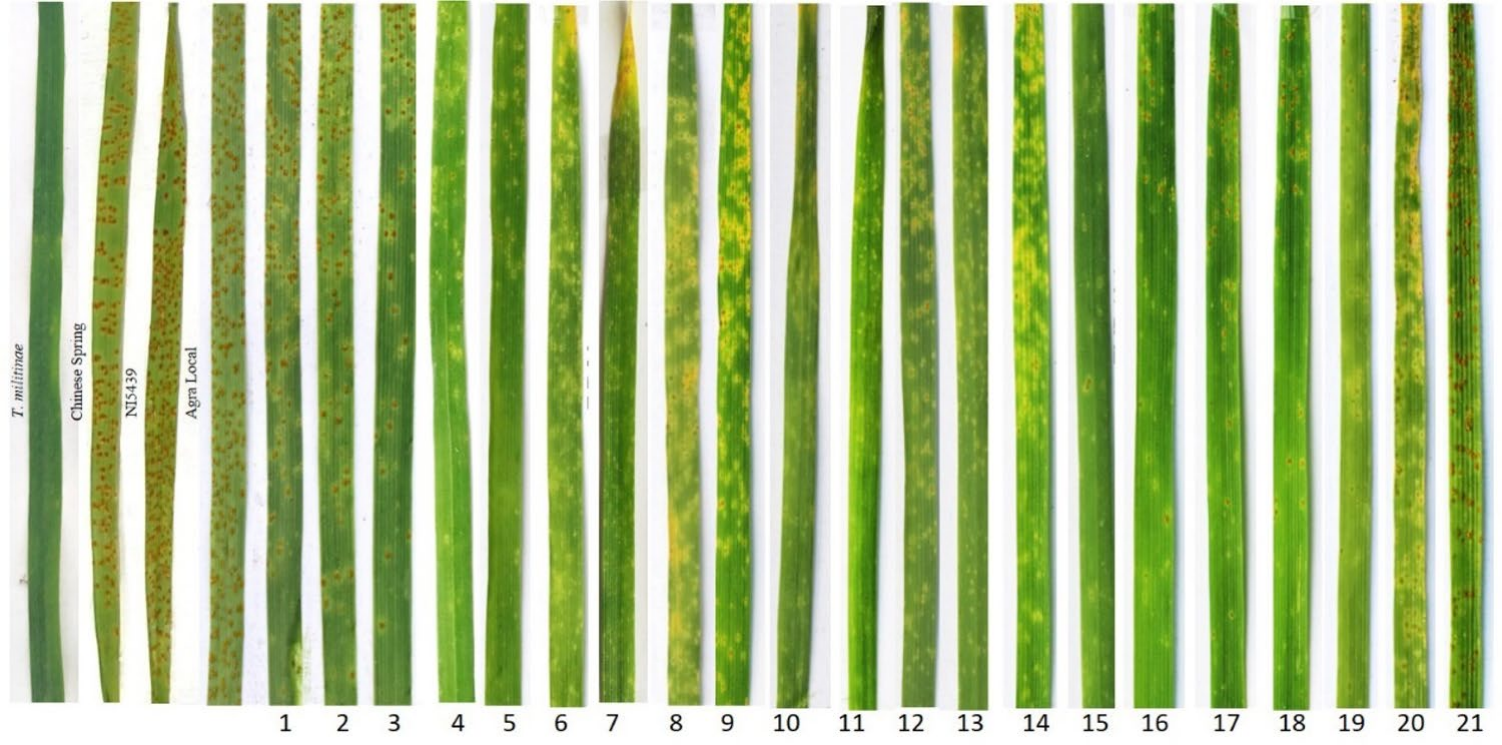
Screening of TMD lines and parental lines against leaf rust race 77–5 at the seedling stage. Here, 1: TMD2-7, 2: TMD2-8, 3: TMD6-1, 4: TMD6-4, 5: TMD6-5, 6: TMD7-4, 7: TMD7-5, 8: TMD9-1, 9: TMD9-2, 10: TMD11-5, 11: TMD11-6, 12: TMD12-4, 13: TMD12-8, 14: TMD12-12, 15: TMD13-1, 16: TMD13-3, 17: TMD13-4, 18: TMD13-7, 19: TMD13-9, 20: TMD15 and 21: TMD17.

Out of 21 lines screened for leaf rust resistance, 17 lines (TMD6-4, TMD6-5, TMD7-5, TMD7-6, TMD9-1, TMD9-2, TMD11-5, TMD11-6, TMD12-4, TMD12-8, TMD12-12, TMD13-1, TMD13-3, TMD13-4, TMD13-7, TMD13-9 and TMD15) (Table 1) showed presence of SR (Seedling Resistant) genes as they were resistant at seedling and adult plant stages. Three lines (TMD2-7, TMD2-8 and TMD6-1) showed the presence of APR (Adult Plant Resistance) genes for leaf rust resistance as they were susceptible at the seedling stage but resistant at the adult plant stage (Table 1). One line, TMD17, was found to be susceptible to leaf rust at both stages. Seedling and adult plant reaction of TMD lines and their parental lines to leaf rust race 77–5 is presented in Fig. 2 and Table 1.

**Table 1:** Pedigree and disease reaction of *T. militinae* derivatives for leaf and stripe rusts.

S. no	Name of the line	Leaf rust		Stripe rust		Pedigree
		Seedling	Adult plant stage	Seedling	Adult plant stage	
*T. militinae* derivatives						
1	TMD2-7	3	R	3+	R	CS/*T.militinae*/ChineseSpring (BC_3_F_8_)
2	TMD2-8	3	R	3+	R	CS/*T.militinae*/ChineseSpring (BC_3_F_8_)
3	TMD6-1	3	R	3+	R	CS/*T.militinae*/NI5439 (BC_3_F_8_)
4	TMD6-4	;1	R	;	R	CS/*T.militinae*/NI5439 (BC_3_F_8_)
5	TMD6-5	;1	R	;	R	CS/*T.militinae*/NI5439 (BC_3_F_8_)
6	TMD7-5	;	R	3	R	CS/*T.militinae*/ChineseSpring (BC_3_F_8_)
7	TMD7-6	;	R	3	MR	CS/*T.militinae*/ChineseSpring (BC_3_F_8_)
8	TMD9-1	;	R	3	S	CS/*T.militinae*/ChineseSpring (BC_3_F_8_)
9	TMD9-2	;12	R	3	S	CS/*T.militinae*/ChineseSpring (BC_3_F_8_)
10	TMD11-5	;	R	3	R	CS/*T.militinae*/ChineseSpring (BC_3_F_8_)
11	TMD11-6	;	MR	;	MR	CS/*T.militinae*/ChineseSpring (BC_3_F_8_)
12	TMD12-4	;1	R	;	R	CS/*T.militinae*/NI5439 (BC_3_F_8_)
13	TMD12-8	;	R	;	R	CS/*T.militinae*/NI5439 (BC_3_F_8_)
14	TMD12-12	;	R	;	MR	CS/*T.militinae*/NI5439 (BC_3_F_8_)
15	TMD13-1	;1	R	3	R	CS/*T.militinae*/NI5439 (BC_3_F_8_)
16	TMD13-3	X	R	3	R	CS/*T.militinae*/NI5439 (BC_3_F_8_)
17	TMD13-4	X	R	3	R	CS/*T.militinae*/NI5439 (BC_3_F_8_)
18	TMD13-7	X	R	;	R	CS/*T.militinae*/NI5439 (BC_3_F_8_)
19	TMD13-9	X	R	;	R	CS/*T.militinae*/NI5439 (BC_3_F_8_)
20	TMD15	X	R	3	S	CS/*T.militinae*/NI5439 (BC_3_F_8_)
21	TMD17	3	S	3	S	CS/*T.militinae*/NI5439 (BC_3_F_8_)
Parental Lines and Susceptible checks						
1	*T. militinae*	0	R	;	R	–
2	Chinese Spring	3	R	3	R	–
3	NI5439	3	S	3	S	–
4	Agra Local	3	S	3	S	–

### Identification of stripe rust resistant *T. militinae* derivatives

**Figure 3:**
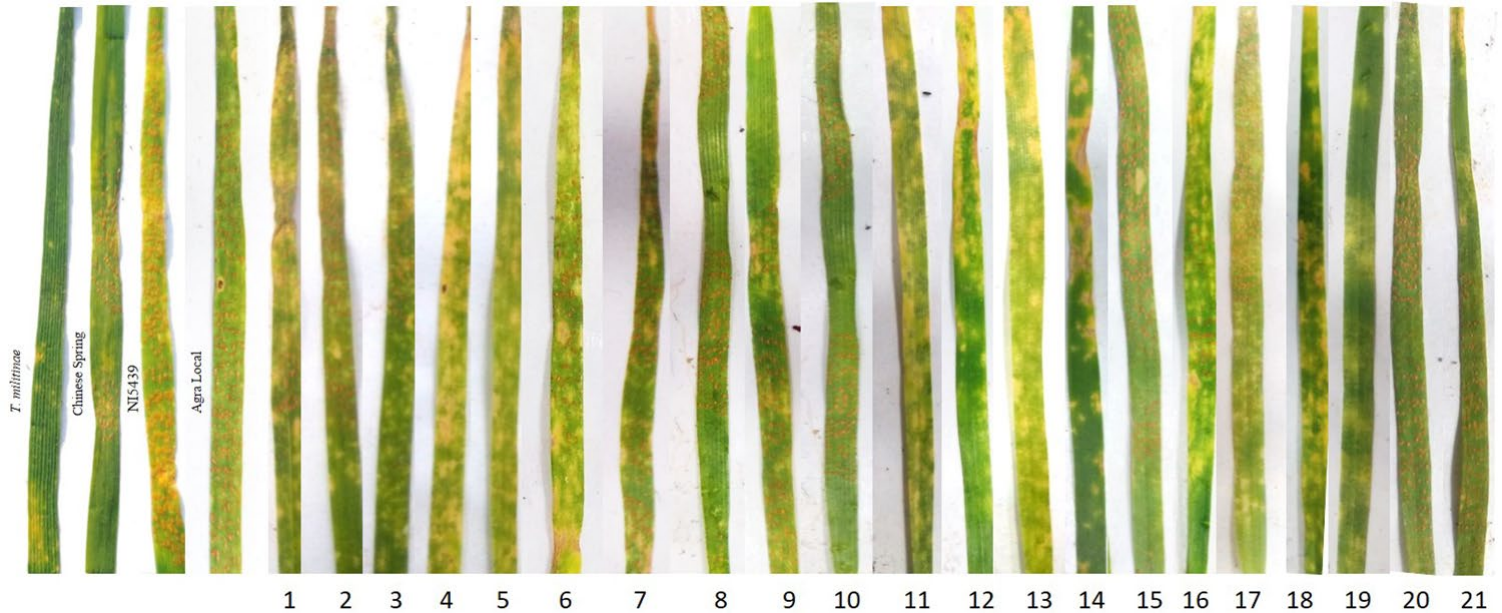
Screening of TMD lines and parental lines against stripe rust race 110S119 at the seedling stage. Here, 1:TMD2-7, 2:TMD2-8, 3:TMD6-1, 4:TMD6-4, 5:TMD6-5, 6:TMD7-4, 7: TMD7-5, 8: TMD9-1, 9: TMD9-2, 10: TMD11-5, 11: TMD11-6, 12: TMD12-4, 13: TMD12-8, 14: TMD12-12, 15: TMD13-1, 16: TMD13-3, 17: TMD13-4, 18: TMD13-7, 19: TMD13-9, 20: TMD15 and 21: TMD17.

Evaluation for stripe rust resistance identified eight lines (TMD6-4, TMD6-5, TMD11-6, TMD12-4, TMD12-8, TMD12-12, TMD13-7 and TMD13-9) with seedling resistance genes and nine lines (TMD2-7, TMD2-8, TMD6-1, TMD7-5, TMD7-6, TMD11-5, TMD13-1, TMD13-3 and TMD13-4) with APR genes. Four lines (TMD9-1, TMD9-2, TMD15 and TMD17) were susceptible to stripe rust at both the stages. Seedling and adult plant reaction of TMD lines, their parental lines and susceptible check Agra Local to stripe rust race 110S119 is presented in Fig. 3, Table 1 and Suppl. Fig. S1.

Eight lines (TMD6-4, TMD6-5, TMD11-6, TMD12-4, TMD12-8, TMD12-12, TMD13-7 and TMD13-9) showed seedling resistance to both leaf and stripe rusts while three lines (TMD2-7, TMD2-8 and TMD6-1) showed adult plant resistance (APR) to both leaf and stripe rusts (Table 1). TMD17 showed susceptibility to both leaf and stripe rusts.

### Screening with markers of known genes in *T. militinae* derivatives

#### Screening of TMD lines for *Lr34/Yr18*

**Figure 4:**
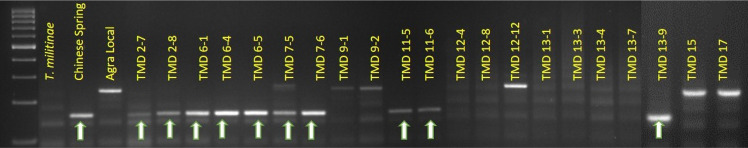
Screening of TMD lines and parental lines for the leaf rust resistance gene *Lr34/Yr18* with the linked marker *csLV34.* Here, lines with arrow marks are positive for the leaf and stripe rust resistance gene *Lr34/Yr18.*

As *T. militinae* derivatives were developed in the background of wheat cultivar Chinese Spring*,* which is known to carry linked genes *Lr34/Yr18* (APR genes for leaf and stripe rust resistance), all the 21 TMD lines were screened with *Lr34* specific marker, *csLV34*^31^. Out of 21 TMD lines, 10 lines showed the presence of *Lr34/Yr18*, while the remaining 11 lines showed its absence (Fig. 4; Table 2). Out of 10, three TMD lines (TMD2-7, TMD2-8 and TMD6-1) were identified as having adult plant resistance to both leaf and stripe rusts, three TMD lines (TMD7-5, TMD7-6 and TMD11-5) with seedling resistance to leaf rust and adult plant resistance to stripe rust and four TMD lines (TMD6-4, TMD6-5, TMD11-6 and TMD13-9) with seedling resistance to both leaf and stripe rusts (Table 1). Out of 10 marker positive TMD lines, six TMD lines (2–7, 2–8, 6–1, 7–5, 7–6 and 11–5) also showed leaf tip necrosis phenotype (phenotypic marker for *Lr34/Yr18*) at adult plant stage (Suppl. Fig. S1).

**Table 2:** Screening of *T. militinae* derivatives with markers of already reported genes from *T. timopheevii*.

S. no	Name of the line	*Lr34/Yr18*	*Lr50*	*Lr18*
*T. militinae* derivatives				
1	TMD2-7	+	+	+
2	TMD2-8	+	+	+
3	TMD6-1	+	+	−
4	TMD6-4	+	−	+
5	TMD6-5	+	+	+
6	TMD7-5	+	−	−
7	TMD7-6	+	−	−
8	TMD9-1	−	−	−
9	TMD9-2	−	−	−
10	TMD11-5	+	−	−
11	TMD11-6	+	−	−
12	TMD12-4	−	−	−
13	TMD12-8	−	−	−
14	TMD12-12	−	−	−
15	TMD13-1	−	+	+
16	TMD13-3	−	+	+
17	TMD13-4	−	−	+
18	TMD13-7	−	+	+
19	TMD13-9		+	+
20	TMD15	−	−	−
21	TMD17	−	−	−
Positive and Negative Checks				
1	*T. timopheevii*	NA	+	+
2	*T. militinae*	−	−	+
3	Chinese Spring	+	−	−
4	Agra Local	−	−	−

Here, ‘+’ means the presence of the gene; ‘−’ means the absence of the gene and ‘NA’ means not amplified.

#### Screening of TMD lines for *Lr18 *and *Lr50*

**Figure 5:**
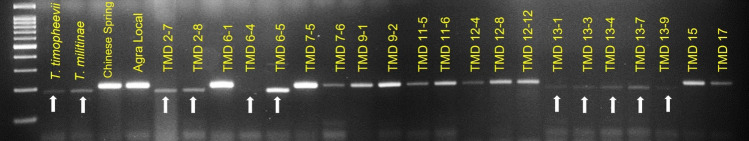
Screening of TMD lines and parental lines for the leaf rust resistance gene *Lr18* with the linked marker *Wmc75.* Here, lines with arrow marks are positive for the gene*.*

Screening of TMD lines with linked marker *Xwmc75*^32^ of *Lr18* suggested the probable presence of *Lr18* in 9 TMD lines (Table 2; Fig. 5). Apart from these, all other TMD lines showed absence of *Lr18* in them. Out of these nine lines, two lines (TMD2-7 and TMD2-8) are identified as having adult plant resistance to both leaf and stripe rusts, four lines (TMD6-4, TMD6-5, TMD13-7 and TMD13-9) with seedling resistance to both leaf and stripe rusts and three lines (TMD13-1, TMD13-3 and TMD13-4) with seedling resistance to leaf rust but adult plant resistance to stripe rusts. *T. militinae* also showed a 180 bp band as of positive check *T. timopheevii* (Fig. 5), indicating the presence of *Lr18* in *T. militinae*.

**Figure 6:**
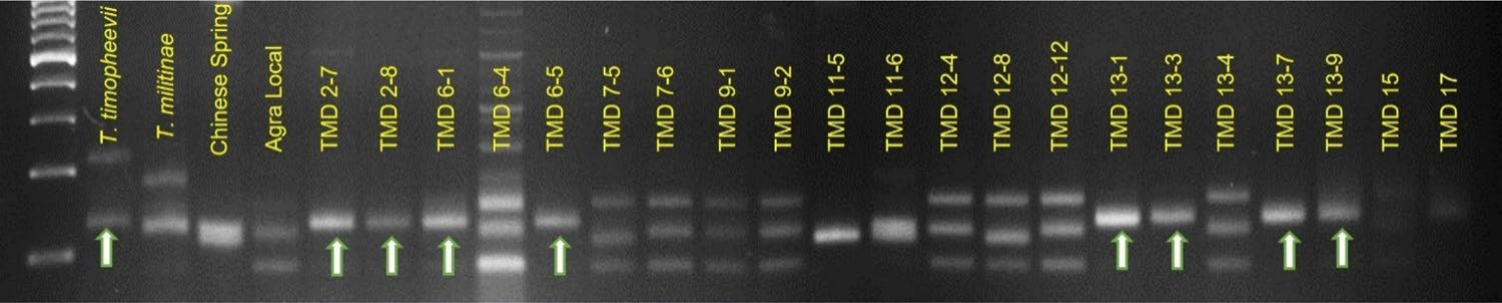
Screening of TMD lines along with parental lines for the leaf rust resistance gene *Lr50* with the linked marker *Xgwm382.* Here, lines with arrow marks are positive for the gene.

Screening with *Xgwm382*^23^ linked marker of *Lr50* suggested the probable presence of *Lr50* in eight *T. militinae* derivatives (Table 2; Fig. 6). Out of these, three lines (TMD2-7, TMD2-8, TMD6-1) are identified of having adult plant resistance to both leaf and stripe rusts, three lines (TMD6-5, TMD13-7 and TMD13-9) with seedling resistance to both leaf and stripe rusts and two lines (TMD13-1 and TMD13-3) with seedling resistance to leaf rusts but adult plant resistance to stripe rusts.

## Discussion

Wild and related species of wheat carry enormous genetic variability that can be utilized in breeding programmes to broaden the genetic base of cultivated wheat. *Triticum militinae,* belonging to the secondary gene pool of wheat, carries enormous genetic variability for disease resistance. To date, no gene that confers rust resistance has been discovered in *T. militinae.* So, characterizing the resistance of *T. militinae* derivatives and knowing their cytological stability is important before using them in inheritance and mapping studies. *T. militinae* (A^t^A^t^GG; 2n = 4X = 28) is a tetraploid with 14 pairs of chromosomes, while *T. aestivum* (AABBDD; 2n = 6X = 42) is a hexaploid with 21 pairs of chromosomes. The transfer of genes from *T. militinae* to *T. aestivum* is possible through homologous and homoeologous pairing. 'G' genome of *T. militinae* has affinity to ‘B’ genome of wheat, which enable it to pair and exchange genomic regions^33^. Crossing of hexaploids with tetraploids produces sterility due to the production of univalents. With repeated backcrossing of F_1_ to hexaploid parent, there is a reduction in the number of univalents and an increase in the number of bivalents, which in turn increases the fertility of backcross derivatives. An increase in fertility levels from BC_1_ to BC_2_ generations is observed in a cross between *T. aestivum* and *Ae. cylindrica*^34^. *T. militinae* derivatives showed 21 bivalents as they have been developed by three generations of backcrossing and seven generations of selfing. The TMD lines with unidentified leaf and stripe rust resistance genes can now be used for inheritance and mapping studies as they are cytologically stable.

Evaluation of *T. militinae* derivatives for leaf and stripe rusts at seedling and adult plant stages and screening with gene based and linked markers of known genes provided insight into the distribution of known leaf rust resistance genes and their resistance pattern in *T. militinae* derivatives. Also, TMD lines with unknown rust-resistance genes were identified for further studies.

Out of seventeen TMD lines identified as carrying seedling resistance to leaf rust, ten TMD lines (7–5, 7–6, 9–1, 9–2, 11–5, 11–6, 12–12, 12–4, 12–8, and 15) found to carry the unknown gene(s) as they showed negative response to markers of known seedling resistance genes *Lr18* and *Lr50* from *T. timopheevii.* Out of ten, four lines (7–5, 7–6, 11–5 and 11–6) showed marker positivity to *Lr34/Yr18,* indicating a known adult plant resistance gene in addition to an unknown seedling resistance gene for leaf rust. The gene *Lr34/Yr18* might have been introduced from Chinese Spring, which was used to develop TMD lines. The presence of *Lr34/Yr18* in addition to *Lr18* in three TMD lines (TMD6-4, TMD6-5 and TMD13-9) is expected to provide added seedling and adult plant resistances^35^.

Rest seven TMD lines with seedling resistance to leaf rust were found to be marker positive for *Lr18*, and some even showed the presence of *Lr50* in them. Leaf rust resistance gene *Lr18* provides seedling resistance to leaf rust but is temperature sensitive. It only provides effective resistance to its avirulent races at lower temperatures (18 °C) and becomes ineffective as the temperature goes beyond 25 °C^36^. So, to confirm the presence of *Lr18*, these lines need to be tested at different temperatures, or it can be confirmed through test of allelism studies. Even the lines that showed the absence of *Lr18* can be tested similarly to confirm its absence. Although identified in some lines, the leaf rust resistance gene *Lr50* is not effective against leaf rust race 77–5 used in the current study. Also, virulence towards *Lr50* existed in races of *P. triticina* even before its deployment in wheat cultivars^23^. So, *Lr50* can provide durable resistance only when it is pyramided along with other effective resistance genes^23^. So, the resistance pattern of TMD lines in the current study is mostly based on seedling resistance gene *Lr18*, adult plant resistance gene *Lr34/Yr18* and/or some unknown genes in *T. militinae*.

Adult plant resistance to leaf and stripe rusts in three TMD lines (TMD2-7, TMD2-8 and TMD6-1) and identification of APR gene *Lr34/Yr18* with marker *csLV34* indicates resistance to leaf and stripe rusts are because of APR genes *Lr34* and *Yr18* respectively. Of the three, two lines, TMD2-7 and TMD2-8, showed the presence of *Lr18* but did not show any seedling resistance to leaf rust. Apart from these two, all other TMD lines (6–4, 6–5, 13–1, 13–3, 13–4, 13–7 and 13–9) identified carrying *Lr18* showed seedling resistance. The susceptibility of lines TMD2-7 and TMD2-8, even with the presence of *Lr18*, may be due to the occurrence of recombination events between the gene *Lr18* and its marker *XWmc75*, present at a distance of 1.2 cM^32^. So, before using any of these 21 TMD lines in further studies, an allelism test with genetic stocks carrying *Lr18* is required.

Screening for stripe rust resistance identified 8 TMD lines with seedling resistance and 9 TMD lines with APR to stripe rust. Out of 9 TMD lines with APR for stripe rust, 6 TMD lines (2–7, 2–8, 6–1, 7–5, 7–6 and 11–5) were found to carry the known gene *Yr18/Lr34* from the wheat cultivar Chinese Spring, used in their development. Three TMD lines (13–1, 13–3 and 13–4) with negative responses to the *Yr18/Lr34* marker indicate the presence of unknown adult plant resistance genes for stripe rust from *T. militinae*. Out of 8 TMD lines with seedling resistance to stripe rust, 4 TMD lines (6–4, 6–5, 11–6 and 13–9) also showed the presence of known APR gene, *Yr18/Lr34* while 4 TMD lines (12–4, 12–8, 2–12 and 13–7) showed its absence. Therefore, all 8 TMD lines may carry unknown/unreported stripe rust resistance gene(s) from *T. militinae*, as till now, not a single stripe rust resistance gene has been reported from both *T. militinae* and *T. timopheevii*. So, the lines with unknown seedling and adult plant resistance genes for stripe rust can be explored soon for mapping and transfer in superior genetic backgrounds. The lines with unknown seedling resistance genes for stripe rust but with known APR gene, *Yr18/Lr34*, can be phenotyped in the seedling stage to tap the seedling stripe rust resistance gene while the other four (without *Yr18/Lr34*) can be tested at both the stages.

Except TMD 17, all other lines were found to carry resistance to either leaf or stripe rusts. This study also highlighted the presence of seedling and adult plant resistance genes in *T. militinae* derivatives, which can be used in future studies for mapping purposes and their utilization in breeding programmes to broaden the genetic base of wheat varieties.

## Conclusion

The present study evaluated 21 *T. militinae* derivatives for leaf and stripe rusts at seedling and adult plant stages. This gave us information about lines carrying seedlings and adult plants' resistance to leaf rust, stripe rust, and leaf and stripe rust. To know whether the resistance in TMD lines is because of known genes or unknown genes, markers of two genes (*Lr18* and *Lr50*) from *T. timopheevii* and one gene (*Lr34/Yr18*) from Chinese Spring were amplified. While ten TMD lines showed the probable presence of unknown genes for leaf rust resistance at the seedling stage, no lines with unknown genes for adult plant resistance to leaf rust could be identified. For stripe rust, while eight lines were identified as carrying unknown genes at the seedling stage, three were identified as carrying unknown genes at the adult plant stage. These lines were also found to be cytologically stable so that they can be utilized in inheritance and mapping studies in future.

## Materials and methods

The plant materials used in the current study are twenty-one derivatives of *T. militinae,* parental lines, *T. militinae* (acc. No. 117001), Chinese Spring and NI5439 used in developing these derivatives and the susceptible check Agra Local. The *T. militinae* acc. no. 117001 was received in 1993 from the Institute of Plant Science Research, Norwich, Norfolk, England, Great Britain. Chinese Spring was used during the screening of TMD lines for leaf and stripe rust resistance and as a positive check for the gene *Lr34/Yr18* during marker screening. The wheat genotypes NI5439, and Agra Local are two susceptible cultivars of India, susceptible to all three types of wheat rusts. While NI5439 and Agra Local were used as susceptible checks during screening for leaf and stripe rusts, only Agra Local was used when screening TMD lines for known markers. All the lines were screened with leaf rust pathotype 77–5 and stripe rust pathotype 110S119. In India, the leaf rust race 77–5 has remained most predominant for more than 20 years, but recently, the leaf rust race 77–9 became wider spread than 77–5^37^. In the case of stripe rust, races 110S119, 238S119 and 46S119 are the three most prevalent and widespread races with a maximum frequency of 110S119 during 2021–22^37^. The race 77–5 is virulent on leaf rust resistance genes *Lr1*, *Lr2a*, *Lr2c*, *Lr3a*, *Lr10*, *Lr13*, *Lr14a*, *Lr15*, *Lr17*, *Lr20*, *Lr23* and *Lr26* and is avirulent on *Lr9*, *Lr18*, *L19*, *Lr24* and *Lr28*. The stripe rust race 110S119 is virulent on stripe rust resistance genes *Yr2*, *Yr2*+, *Yr3*, *Yr4*, *Yr6*, *Yr7*, *Yr8*, *Yr9*, *Yr6*(un) *Yr7*(un) and avirulent on *Yr1*, *Yr5*, *Yr9* + and *Yr10*. To identify new sources of leaf rust resistance, markers of designated leaf rust resistance genes, *Lr18* and *Lr50* from *T. timopheevii,* were screened in TMD lines, where *T. timopheevii* was used as a positive control. TMD lines were also screened with *Lr34/Yr18* markers, as the Chinese Spring was used as a parental line during their development. TMD lines were screened for leaf and stripe rusts at seedling and adult plant stages. As most of the TMD lines showed resistance to either leaf, stripe or both diseases, their cytological stability was also studied at the meiotic metaphase stage so that they can be used in inheritance and mapping studies. The list of TMD lines used in the current study and their pedigree is given in Table 1. All methods were performed following the relevant guidelines/regulations/legislation.

### Analysis of cytological stability of *T. militinae* derivatives

To study the cytological stability of *T. militinae* derivatives, immature spikes of appropriate size were collected during the booting stage. Anthers containing pollen mother cells (PMCs) at meiotic metaphase I were identified, fixed in Carnoy’s I fixative, and stored at 4 °C. For slide preparation, the anthers were hydrolyzed in a 1N HCl solution at 60 °C for 12 min. Following this, they were stained with Feulgen solution in the dark. Subsequently, slides were prepared using the squash method, utilizing 2% acetocarmine solution.

### Screening for leaf and stripe rust resistance at the seedling stage

Screening for leaf and stripe rust resistance was carried out with pathotypes 77–5 and 110S119, respectively, in the glass house of the Division of Genetics. The screening experiments were repeated during 2018–19 and 2019–20. The screening materials included 21 TMD lines, their parental lines, *T. militinae*, Chinese Spring and NI5439 and the susceptible check Agra Local (AL). All the lines were sown in aluminium trays of 4 × 10 × 3 inches in size in the glass house at the Division of Genetics, IARI, New Delhi. Seedlings of ten days old with completely opened primary leaves were inoculated separately with leaf and stripe rust races. An inoculation mixture of concentration ~6 × 10^5^ spores/ml was prepared by adding urediospores in tap water. A drop of tween20 was added to the inoculation mixture to attain affinity and uniform spread of the urediospores on the leaf surface. After inoculation, the trays were kept in the incubation chamber under diffused light for 48 h and then shifted to glass house benches. Infection types on each introgression line were recorded 12–14 days after inoculation, following the standard scoring method^38^.

### Screening for leaf and stripe rusts at the adult plant stage

For screening of TMD lines at the adult plant stage, all 21 TMD lines, along with their parental lines, were sown in leaf and stripe rust nurseries. In the rust nursery, each line was planted in 1 m rows, with infector rows planted after every 20 lines. Two rows of infectors were also planted in borders to have sufficient and uniform disease spread. Spores of leaf rust race 77–5 and stripe rust race 110S119 were sprayed in the respective rust nurseries as a suspension in water fortified with Tween20 (0.75 µl/ml) at an average concentration of 20 urediospores/microscopic field (10x × 10x). Inoculum suspension was also injected into the last internode of the plant with the help of a 2 ml hypodermic syringe at the boot leaf stage in the field before the emergence of the boot^39^. Plant response was recorded into five infection types^40^.

### Screening with markers of known genes in *T. militinae* derivatives

To know whether these TMD lines carry known or unknown rust resistance genes, markers of leaf rust resistance genes *Lr18* and *Lr50* were used to screen TMD lines. Here, *T. timopheevii* was used as a positive check for *Lr18* and *Lr50* and Agra Local and NI5439 were used as negative checks. Though, no stripe rust resistance gene was reported from *T. timopheevii* or *T. militinae*, a marker of *Yr18/Lr34* was also used in screening, as Chinese Spring was used as one of the parents in the development of TMD lines, which is known to carry linked stripe and leaf rust resistance gene *Yr18/Lr34*. Details of molecular markers used in the screening are presented in Table 3.

**Table 3:** Details of markers used in screening of *T. militinae* derivatives.

Rust resistance gene	Chromosome	Linked Molecular marker	Distance from the gene (cM)	Product size (bp)
*Lr18*	5BL	*XWmc75*	1.2	180
*Lr50*	2BL	*Xgwm382*	6.5	139
*Yr18/Lr34*	7DS	*csLV34*	0.4	150

### Supplementary Information

Supplementary Information.

## Data Availability

The datasets used and/or analyzed during the current study are available from the corresponding author on reasonable request. All methods were performed in accordance with the relevant guidelines/regulations/legislation by including a statement in the manuscript to this effect?
