# Supplementary material for: Characterization and identification of sources of rust resistance in Triticum militinae derivatives
Source: Sci Rep. 2024 Apr 24;14:9408. doi: 10.1038/s41598-024-59902-x (PMC11043457; doi:10.1038/s41598-024-59902-x)

**Characterization and identification of sources of rust resistance in *Triticum militinae* derivatives**

**Saikat Chowdhury^1,3^, Shreshtha Bansal^1,3^, Shailendra K. Jha^1^, M.S. Saharan^2^, Niranjana, M^1^., Raghunandan K^1^., Manish K. Choudhary^1^, Priyanka Agarwal^1^, Niharika Mallick^1^* and Vinod^1*^**

**^1^Division of Genetics, ICAR-Indian Agricultural Research Institute, New Delhi-110012**

**^2^Division of Plant Pathology, ICAR- Indian Agricultural Research Institute, New Delhi-110012**

**^3^These authors contributed equally: Saikat Chaudhary and Shreshtha Bansal**

**^*^Corresponding Author’s email:** [**niharikamallick@gmail.com**](mailto:niharikamallick@gmail.com)**, vinod.genetics@gmail.com**

**Supplementary Information1:** **Supplementary Figure S1 online**

**
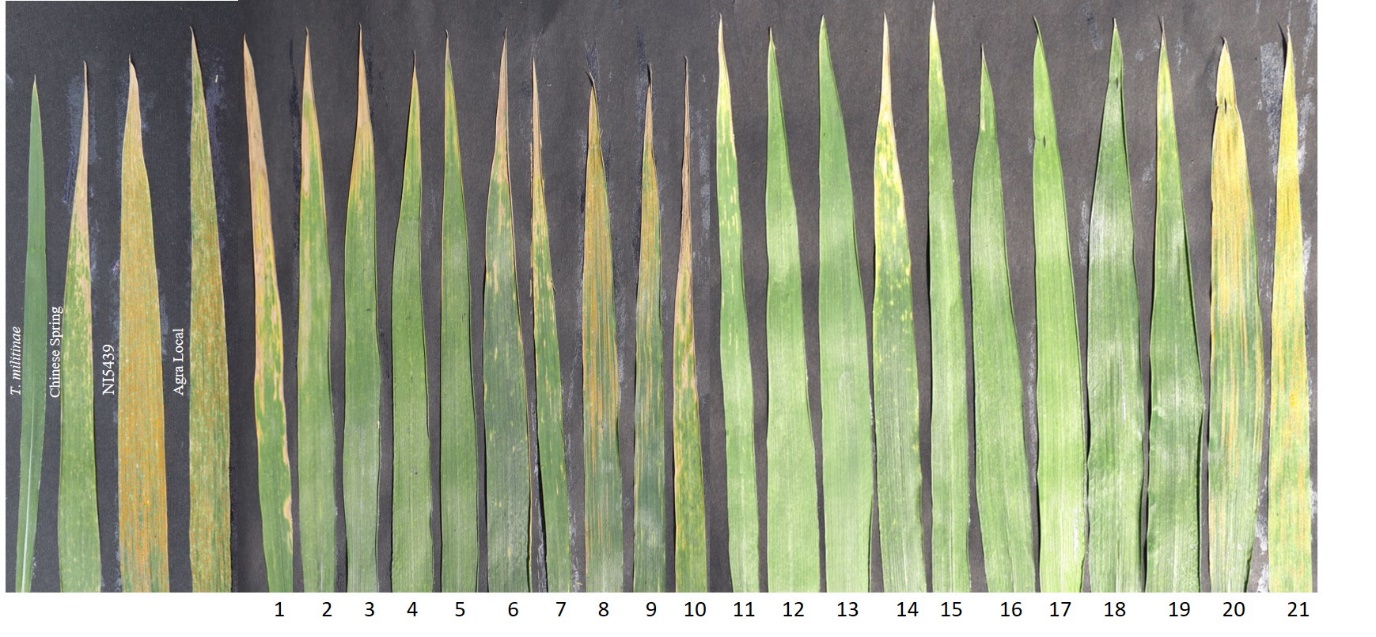
**

**Supplementary Figure S1.** Screening of TMD lines along with parental lines against stripe rust race 110S119 at adult plant stage. Here, 1:TMD2-7, 2:TMD2-8, 3:TMD6-1, 4:TMD6-4, 5:TMD6-5, 6:TMD7-5, 7: TMD7-6, 8: TMD9-1, 9: TMD9-2, 10: TMD11-5, 11: TMD11-6, 12: TMD12-4, 13: TMD12-8, 14: TMD12-12, 15: TMD13-1, 16: TMD13-3, 17: TMD13-4, 18: TMD13-7, 19: TMD13-9, 20: TMD15 and 21: TMD17.

**Supplementary Information 2**: Full-length gels of Figure 4 (a-b) online. Red squares are marking cropped parts of gels presented in the Figure 4.

Figure 4 (a):


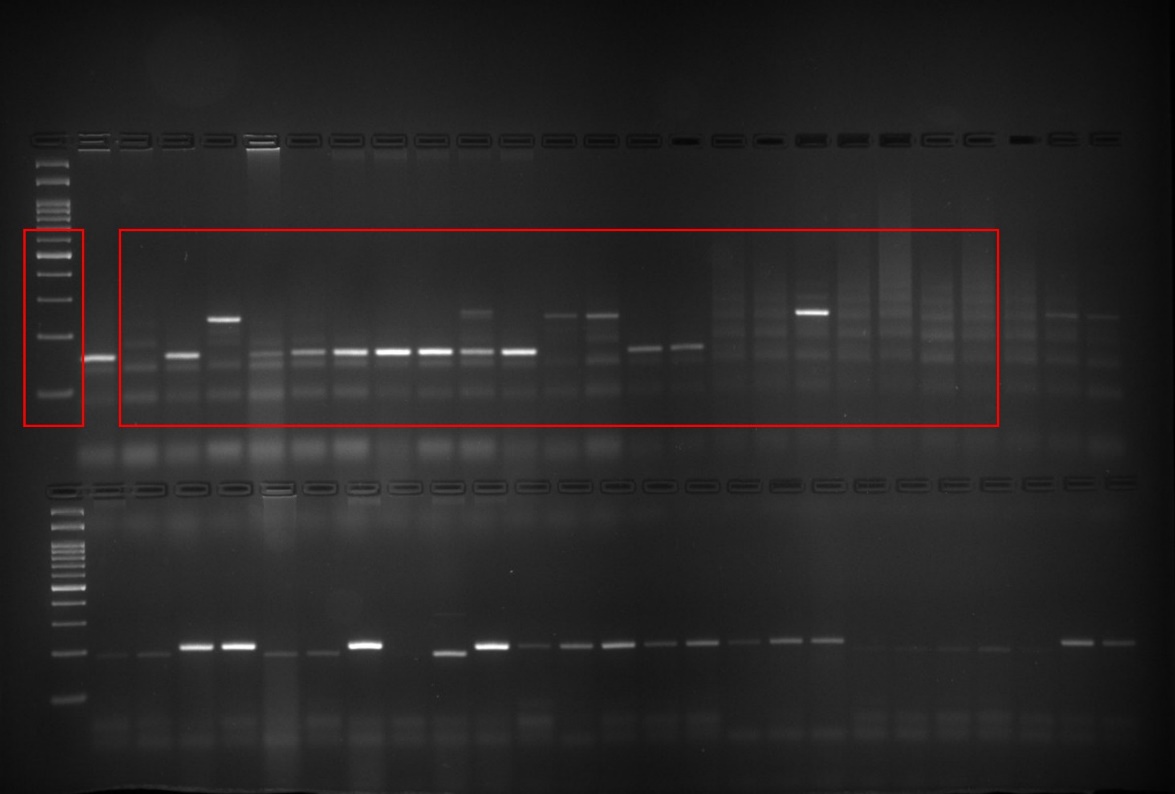


Figure 4 (b):


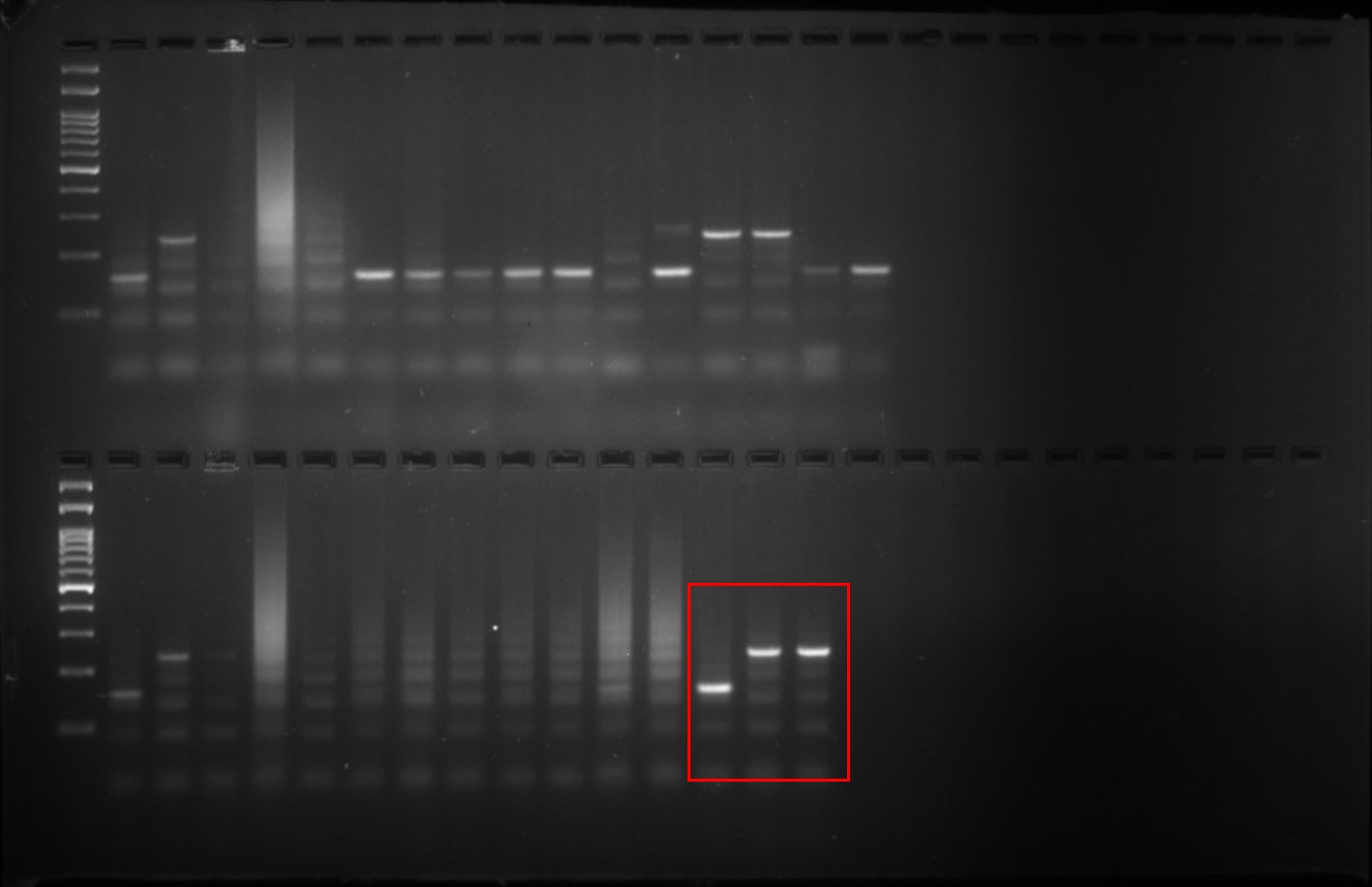


**Supplementary Information 3**: Full-length gel of Figure 5 online. Red square is marking cropped part of gel presented in the Figure 5.


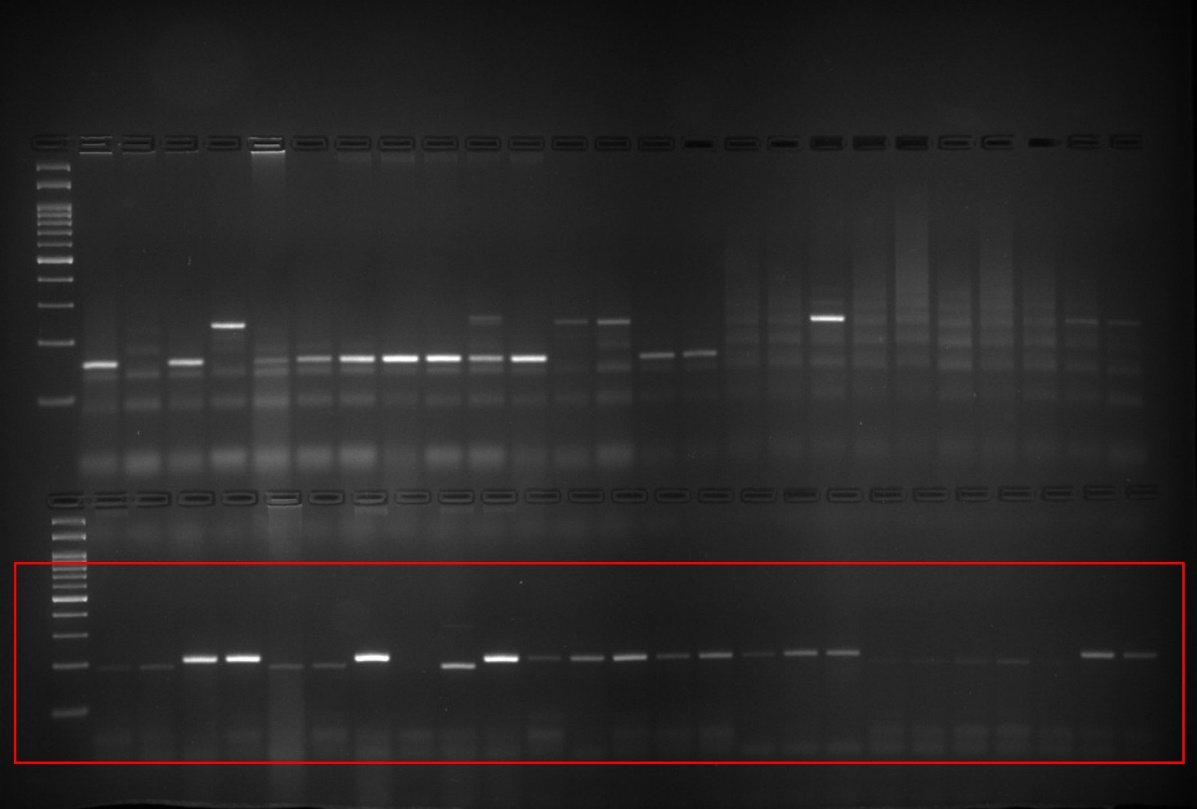


**Supplementary Information 4**: Full-length gel of Figure 6 online. Red square is marking cropped part of gel presented in the Figure 6.


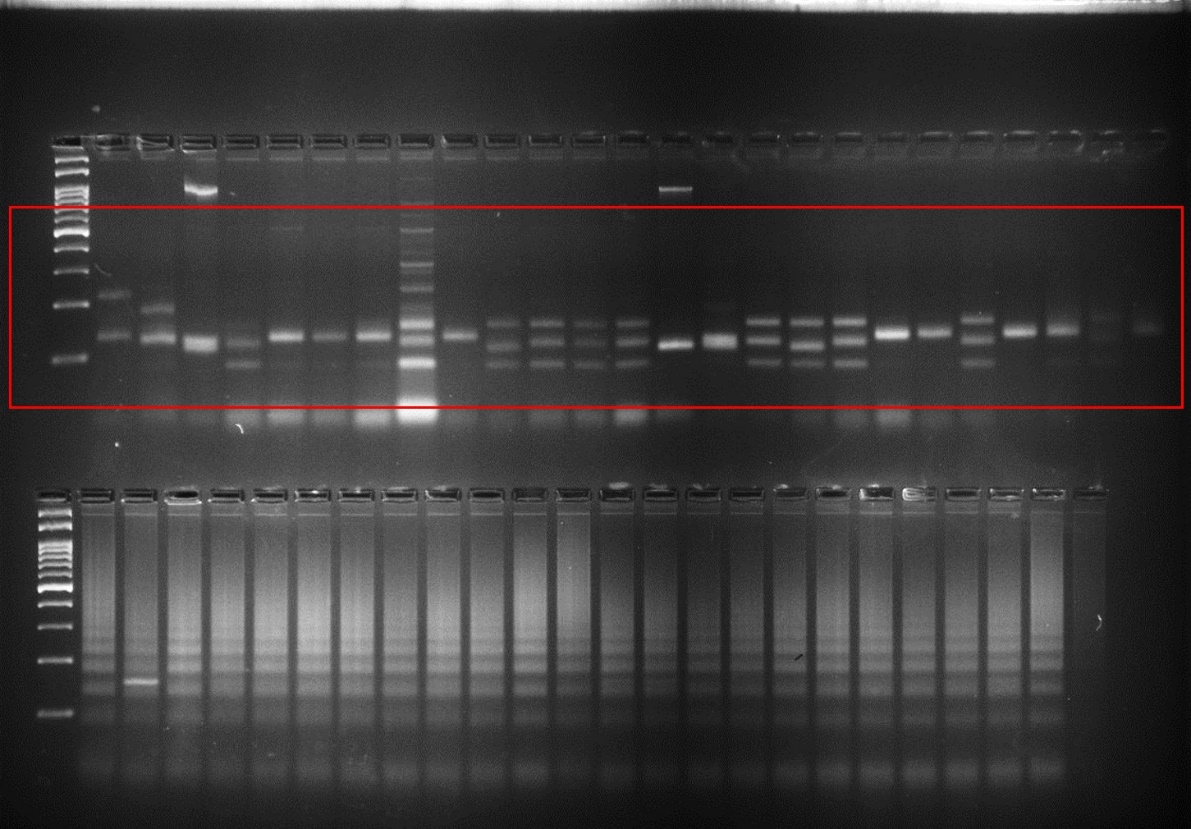

Supplement: Supplementary file 1 — Supplementary Information. [file 41598_2024_59902_MOESM1_ESM.docx]
